# Activation of Human T-Helper/Inducer Cell, T-Cytotoxic Cell, B-Cell, and Natural Killer (NK)-Cells and induction of Natural Killer Cell Activity against K562 Chronic Myeloid Leukemia Cells with Modified Citrus Pectin

**DOI:** 10.1186/1472-6882-11-59

**Published:** 2011-08-04

**Authors:** Cheppail Ramachandran, Barry J Wilk, Arland Hotchkiss, Hoa Chau, Isaac Eliaz, Steven J Melnick

**Affiliations:** 1 Dharma Biomedical LLC, Miami, FL, 33156, USA; 2 Econugenics, Inc., Santa Rosa CA, 95407, USA; 3 U.S. Department of Agriculture, USA; 4 Agricultural Research Service, Eastern Regional Research Center, 600 E. Mermaid Lane, Wyndoor, PA 19038, USA; 5 Department of Pathology, Miami Children's Hospital, Miami, FL 33155, USA

## Abstract

**Background:**

Modified citrus pectin (MCP) is known for its anti-cancer effects and its ability to be absorbed and circulated in the human body. In this report we tested the ability of MCP to induce the activation of human blood lymphocyte subsets like T, B and NK-cells.

**Methods:**

MCP treated human blood samples were incubated with specific antibody combinations and analyzed in a flow cytometer using a 3-color protocol. To test functionality of the activated NK-cells, isolated normal lymphocytes were treated with increasing concentrations of MCP. Log-phase PKH26-labeled K562 leukemic cells were added to the lymphocytes and incubated for 4 h. The mixture was stained with FITC-labeled active form of caspase 3 antibody and analyzed by a 2-color flow cytometry protocol. The percentage of K562 cells positive for PKH26 and FITC were calculated as the dead cells induced by NK-cells. Monosaccharide analysis of the MCP was performed by high-performance anion-exchange chromatography with pulse amperometric detection (HPAEC-PAD).

**Results:**

MCP activated T-cytotoxic cells and B-cell in a dose-dependent manner, and induced significant dose-dependent activation of NK-cells. MCP-activated NK-cells demonstrated functionality in inducing cancer cell death. MCP consisted of oligogalacturonic acids with some containing 4,5-unsaturated non-reducing ends.

**Conclusions:**

MCP has immunostimulatory properties in human blood samples, including the activation of functional NK cells against K562 leukemic cells in culture. Unsaturated oligogalacturonic acids appear to be the immunostimulatory carbohydrates in MCP.

## Background

Pectin is a complex carbohydrate soluble fiber. Dietary fibers, such as pectin, have been shown to have positive effects on a wide spectrum of pathological conditions. Their positive influence on human health is explained by their anti-oxidative, hypocholesterolemic, and anti-cancer effects [1-12]. The effect on the immune system has been previously attributed to the down regulation of the inflammatory response, moderating the production of pro inflammatory cytokines and immunoglobulins in murine models for irritable bowel syndrome [13]. A diet rich in soluble fiber in an animal model showed protection against endotoxin-induced sickness behavior by cytokine modulation and promotion of alternative activation of macrophages [14]. Citrus pectin has the capacity to exert a favourable immunomodulatory response in human peripheral blood cells through its effect on cytokine production [15]. High methoxy citrus pectin inhibits the binding of fibroblast growth factor-1 (FGF-1) to its receptor in the presence of heparin [16]. The rhamnogalacturonan I-arabinan fraction of pectin from a medicinal herb enhances secretion of granulocyte colony-stimulating factor (G-CSF) by murine colonic MCE 301 cells [17]. Rhamnogalacturonan I-arabinogalactan was also reported to activate macrophages and dendritic cells [18]. Methyl-esterified pectic oligosaccharides with 4,5-unsaturated non-reducing ends enhanced T-helper1 (Th1) dependent delayed-type hypersensitivity in a murine influenza vaccine model, reduced Th2 cytokine (IL-4, IL-5 and IL-10) production in splenocytes *in vitro *[19] and decreased allergic asthma in mice [20]. Therefore, the carbohydrate composition of pectin is very important in determining different immune responses.

The modified citrus pectin (MCP) used in this study, is composed of short, slightly-branched, carbohydrate chains derived from the soluble albedo fraction of citrus fruit peels altered by decreasing the molecular weight and degree of esterification using pH, temperature and a controlled enzymatic process, in order to increase its absorption into the circulatory system. MCP is relatively rich in galactose, and antagonizes a binding protein galectin-3 (Gal-3), which results in suppression of cancer cell aggregation, adhesion, and metastasis [5,6]. MCP acts as a ligand for Gal3, which plays a major role in tumor formation and progression [12,21-24]. It has been shown using a combination of fluorescence microscopy, flow cytometry, and atomic force microscopy, that specific binding of a pectin galactan to the recombinant form of human galectin-3 has been physically observed [25]. Moreover, MCP also showed anti-metastatic effects on cancer cells *in vitro *or *in vivo *[8,10,11,24,26-28]. Human clinical trials with MCP showed an increase in prostate specific antigen doubling time, a marker of slowing the progression of prostate cancer [9], and significant improvement of quality of life and stabilization of disease for patients with advanced solid tumors [29]. Besides the therapeutic roles against cancer, MCP has been shown to remove toxic metals from the body [30,31], and reduce experimentally induced kidney injury and fibrosis *in vivo *by reducing galectin-3 levels [32]. In the United States of America, MCP is registered as a food supplement and is generally regarded as safe (GRAS).

*In vitro *lymphocyte activation represents a standard approach for evaluating cell-mediated responses to a variety of stimuli including immunostimulatory botanical extracts. An appropriate assay system monitors the expression of the early activation marker CD69 in whole blood after stimulation with extracts. CD69 is expressed in all activated lymphocyte subsets and hence it represents a generic marker to monitor individual subset responses to specific stimuli [33]. CD4 antigen is expressed on the T- helper/inducer lymphocyte subset (CD3/CD4). CD8 antigen is expressed on the human cytotoxic T-lymphocyte subset (CD3/CD8). Once activated both CD4 and CD8 positive T cells express CD69. T-lymphocyte subsets can be identified and quantified by using fluorochrome-labelled antibody combinations such as CD4/CD69/CD3 and CD8/CD69/CD3. CD19 antigen is present on human B-lymphocytes at all stages of maturation and is not present on resting or activated T-lymphocytes. CD19/CD69/CD45 labelled antibody combination can be used to identify an activated B cell population. CD56 antigen is present on Natural Killer (NK)-cells and antigen intensity increases with NK-cell activation. Hence, CD56/CD69/CD45 labelled antibody combination can be used to identify activated NK-cells. The ability of NK-cell subset in normal lymphocytes to induce death in leukemia cells is analyzed by co-incubating MCP treated lymphocytes with K562 T-cell leukemia cells. In this report we tested the ability of MCP to induce the activation of human blood lymphocyte subsets (T-helper/inducer, T-cytotoxic, B-cell, and NK-cell), and the induced NK-cell's activity against K562 chronic myeloid leukemia cells. Carbohydrate composition analysis was also performed to propose a structural mechanism of action of this immune enhancement.

## Methods

MCP (PectaSol-C^® ^MCP, Econugenics, Inc., Santa Rosa, CA, USA) was initially solubilized in phosphate buffered saline (PBS). The solubility of MCP in PBS was 76.4%. The volume was adjusted to get accurate amounts of the compound for treatment based on solubility factor.

T-, B- and NK-cell activation assay: Blood samples were collected from three unidentified healthy volunteers based on an exempt study protocol submitted and approved by the Miami Children's Hospital Institutional Review Board. Blood samples (250 μl) were incubated in 48-well plates with increasing concentrations of compound along with appropriate positive controls (recommended by Becton Dickinson Biosciences, CA, USA) for each subset. CD2/CD2R and Phorbol ester (PMA) were used as controls for T-cytotoxic cell activation studies. Pokeweed (PWM) mitogen was used as positive control for B-cell activation and IL-2 was used for activating NK-cells in blood cultures. The blood cultures were incubated at 37°C in a CO_2 _incubator for 24 h. On the next day, 20 μl of specific antibody mix, [CD4-FITC/CD69-PE/CD45-PerCP (T-helper/inducer cell activation), CD8-FITC/CD69-PE/CD3-PerCP (T-cytotoxic cell activation), CD19-FITC/CD69-PE/CD45-PerCP (B-cell activation) and CD56-FITC/CD69-PE/CD45-PerCP (NK-cell activation)] was dispensed into separate flow tubes (in duplicate) and 50 μl blood sample was mixed with antibody and incubated for 30 min at room temperature in the dark. The blood-antibody mix was lyzed in a Coulter Epics Q-prep work station using Immunoprep kit and run on a Coulter Elite flow cytometer using a 3-color protocol. The percentage of activated T-helper cells, T-cytotoxic cells, B-cells and NK-cells and the percentage increase over untreated control were calculated and plotted against compound concentrations.

Functional NK Cell Activity Assay: Normal lymphocytes were isolated from three healthy volunteers using histopaque 1077 gradient centrifugation. The cells were washed twice with PBS and re-suspended in phenol-free RPMI 1640 medium supplemented with 10% fetal bovine serum and antibiotics (complete medium). Cells were plated in special 48-well deep plates (conical bottom) at 10^6 ^cells/ml/well and treated with increasing concentrations (0-800 ug/ml) of MCP in a 5% CO_2 _incubator at 37°C for 24 h. On the next day, log-phase K562 leukemic cells were labelled with PKH26 membrane dye (Sigma, St. Louis, MO, USA) for 5 min according to manufacturer's protocol and 0.2 × 10^6 ^cells each were added to the normal lymphocytes in the 48 well plates. The plates were centrifuged for 1 min at 250 × g and returned to the CO_2 _incubator for another 4 hours for inducing cell death.

The cell mixture was permeabilized by incubating in 2% paraformaldehyde solution at 4°C followed by incubation in 0.2% Tween 20 solution (PBS) at 37°C. The cell mixture was washed with ice-cold PBS and stained with human specific FITC-labeled active form of caspase 3 antibody for 30 minutes at room temperature according to the procedure published earlier [34,35]. The stained cells were washed with 0.1% Tween 20 solution, resuspended in 0.5 ml of staining buffer and analyzed by a two-color flow cytometry protocol with FL1 and FL2 measuring PKH26 and FITC, respectively in a Beckman Coulter Elite Flow cytometer. The percentage of K562 cells positive for PKH26 and FITC are dead cells induced by NK-cells.

Statistical Analysis: Mean and standard deviation values were calculated and graphs prepared by Microsoft Excel. Kruskal-Wallis one-way Analysis of Variance (ANOVA) was used to analyze the data by the GraphPad Prism 5 software and *p *values were estimated. Multiple comparisons of treatments in all possible combinations were performed by Tukey-Kramer post test using the GraphPad Prism 5 software.

Carbohydrate Composition Analysis of MCP: Monosaccharide analysis was performed by high-performance anion-exchange chromatography with pulse amperometric detection (HPAEC-PAD) following methanolysis according previously published method [36]. HPAEC-PAD was also used for oligosaccharide analysis [32]. Weight average intrinsic viscosity analysis was performed by high-performance size-exclusion chromatography (HPSEC) with multiple detectors (multi-angle laser light scattering, refractive index and differential pressure viscometer according to previously publish methods [30].

## Results

### T-Lymphocyte Subset Activation

**Figure 1:**
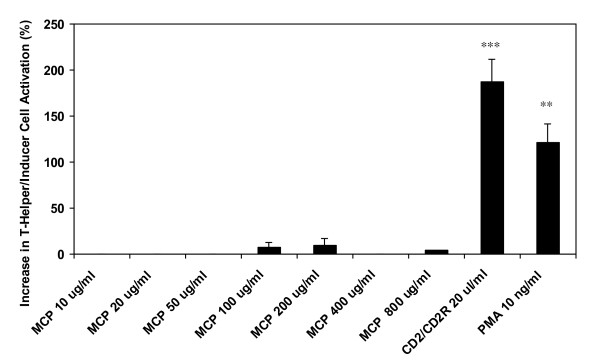
**Increase in T-Helper/Inducer Cell activation (%) by MCP**. (**p < 0.01, ***p < 0.001).

**Figure 2:**
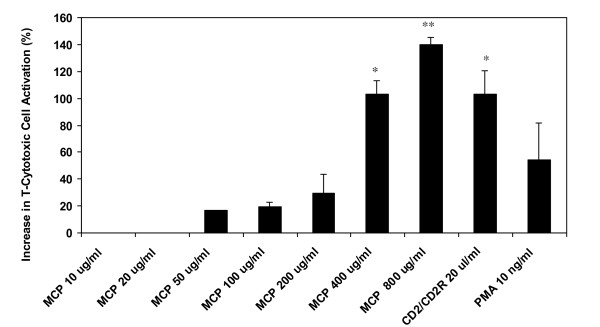
**Increase in T-Cytotoxic Cell activation (%) by MCP**. (*p < 0.05, **p < 0.01).

The percent increases in T- helper/inducer lymphocyte and T-cytotoxic cell activation are shown in Figure 1 &2, respectively. All positive controls induced expected responses. Results show that MCP does not have a significant effect on T-helper/inducer cell activation as compared to positive controls like CD2/CD2R (*p *< 0.01) and PMA (*p *< 0.001). However, MCP activated T-cytotoxic cells at lower levels and in a dose-dependent manner between 50-800 ug/ml concentrations. MCP-induced T-cytotoxic cell activation was significant at 400 ug/ml (*p *< 0.05) and highly significant at 800 ug/ml (*p *< 0.01) concentrations.

### B-Cell Activation

**Figure 3:**
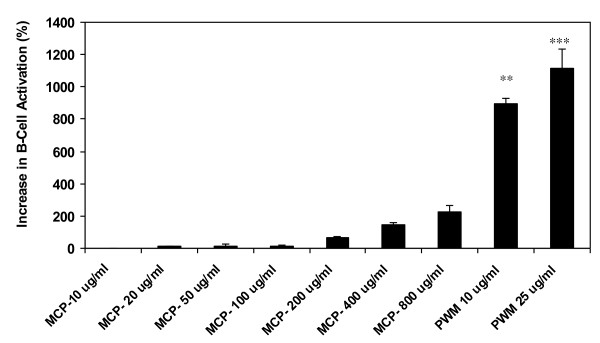
**Increase in B-Cell activation (%) by MCP**. (**p < 0.01, ***p < 0.001).

The percentage increase of MCP on B-cell activation over untreated control is given in Figure 3. The positive control PWM induced highly significant activation at 10 ug/ml (*p *< 0.01) and 25 ug/ml (*p *< 0.001) concentrations. MCP induced a dose-dependent activation of B-cells, although the level of activation was lower than that of PWM and not significant by Tukey-Kramer test.

### NK-Cell Activation

**Figure 4:**
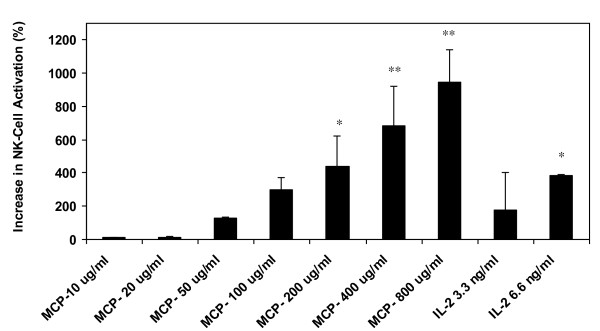
**Increase in NK-Cell activation (%) by MCP**. (*p < 0.05, **p < 0.01).

The effects of MCP on the percentage increase of NK-cell activation over untreated control is given in Figure 4. The positive control IL-2 at 6.6 ng/ml induced a significant level of NK-cell activation (*p *< 0.05). MCP demonstrated a dose-dependent activation of NK-cells with a lower significance level (*p *< 0.05) attained at 200 ug/ml and a highly significant level at 400 and 800 ug/ml concentrations (*p *< 0.01).

### MCP activated NK-Cell activity on K562 Chronic Myeloid Leukemia Cells

**Figure 5:**
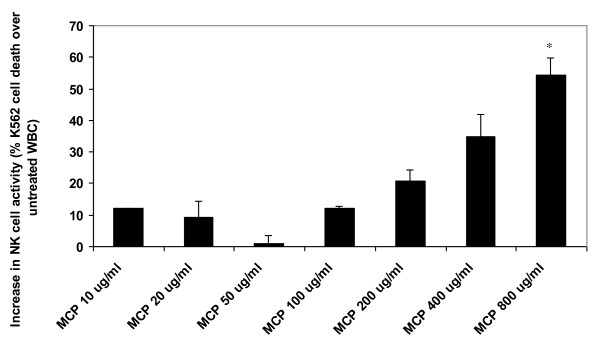
**Increase in Percent NK-Cell activity by MCP**. (*p < 0.05).

The results of MCP-treated blood samples on increase in percent of functional NK-cell activity is shown in Figure 5. MCP induces NK-cell activity in a dose-dependent manner with 800 ug/ml dose causing about 53.60% increase in NK-cell activity (*p *< 0.05). The dose-dependent increasing trend in NK-cell activity apparently corresponds with the activation of NK-cells obtained by staining with NK-cell activation markers (CD56/CD69/CD45).

### MCP Carbohydrate Composition Analysis

**Figure 6:**
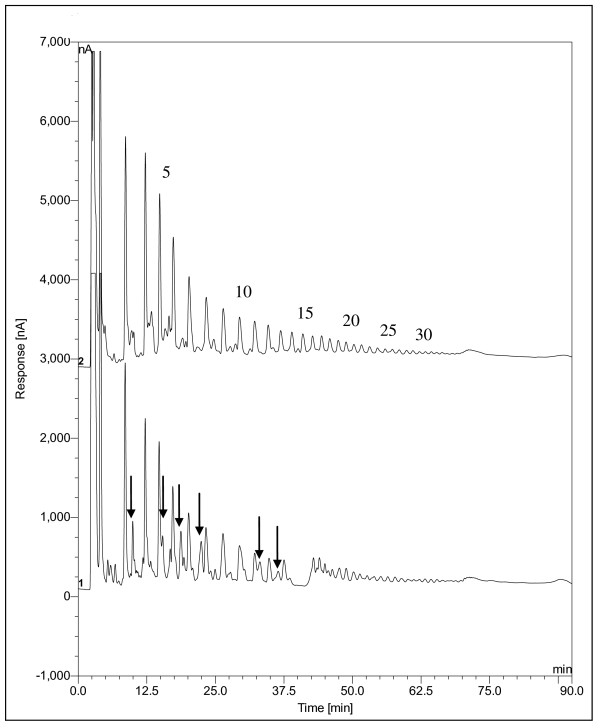
**HPAEC-PAD analysis of MCP (1) compared to a polygalacturonic acid hydrolysate (2)**. The degree of polymerization is listed over the peaks. The arrows point to unsaturated oligogalacturonic acids.

The monosaccharide composition of the MCP was identified and is reported in Table 1. The MCP consisted of mainly galacturonic acid with galactose and arabinose, the most common neutral sugars. The MCP had a high amount of homogalacturonan compared to rhamnogalacturonan (high GalA/Rha ratio). On average the homogalacturonan backbone of the MCP was interrupted with a rhamnose residue once every 22 galacturonic acid residues (Table 1). Arabinan, galactan or arabinogalactan neutral sugar side-chains in pectin are attached to the rhamnose residues in rhamnogalacturonan. The homogalacturonan consisted of both saturated and unsaturated oligogalacturonic acids (Figure 6). The unsaturated oligogalacturonic acids had longer retention times compared to saturated oligogalacturonic acids [36]. The MCP average molar mass (3,320 Da), average intrinsic viscosity (0.0648 dL/g), and Mark Houwink constant (*a *= 0.824) are reported (Table 2).

**Table 1:** MCP Monosaccharide Composition (Mole %)

Monosaccharide	Mole %
Glucose	2.17
Arabinose	3.28
Galactose	10.31
Xylose	1.45
Rhamnose	3.53
Fructose	0.13
Galacturonic Acid	79.03
Glucuronic Acid	0.11
Galacturonic Acid/Rhamnose	22.39

**Table 2:** MCP Average Molecular Weight, Intrinsic Viscosity and Shape Analysis (Mark Houwink Constant)

Average Molar Mass (Da)	Average Intrinsic Viscosity (dL/g)	Mark Houwink Constant (*a*)
3,320 ± 300	0.0648 ± 0.001	0.824 ± 0.03

Values are mean ± SD

## Discussion

The immune system protects people from disease causing microorganisms and other harmful materials. Circulating blood transports immune components between organs of the immune system and sites of inflammation. The enumerative (activation marker assays) and functional assays (NK cell activity assay) used in the present study to evaluate the immunostimulative potential of MCP clearly demonstrated activation of T cytotoxic and NK cells in blood cultures *in vitro*. Furthermore, functional assay indicated that MCP enhanced cytolysis of leukemic cells by NK cells. It is well known that NK cell activity is regulated by the expression levels of cytotoxic molecules, activating receptors and/or inhibitory receptors. Even though several CAM agents have been shown to activate NK-cells and improve NK cell activity, the precise mechanism is not clearly defined. The two main possibilities are: (i) augmentation of cytotoxic molecules in NK cells and/or (ii) up-regulation of activating NK receptors and/or down-regulation of inhibitory NK receptors [37,38].

The mechanism for selective immunostimulation of T cytotoxic and NK cells by MCP can only be speculated. It is suggested that, except for a lower degree of esterification, the MCP pectic oligosaccharide composition and molecular weight is quite similar to other pectic oligosaccharides reported to polarize the T-helper response toward Th1 in a murine influenza vaccination model [19] and those used in subsequent studies of murine allergic asthma [20] and human infant formula feeding [39]. Therefore, unsaturated pectic oligosaccharides appear to be required for immunostimulation and a relatively high degree of esterification may be necessary for stimulation of T-helper cells.

The average intrinsic viscosity of pectin is directly proportional to the molar mass, thus demonstrating a lower molecular weight range of the MCP compared to 14,800 Da and 0.398 dL/g reported previously [30]. The lower Mark Houwink constant for MCP compared to the 1.45 value that we observed previously [30] means that the global structure has more random coil shape typical of citrus pectin, while a higher Mark Houwink constant (*a*) was interpreted as being more rigid and rod like. Therefore, MCP is a flexible low molecular weight pectin polymer enriched in saturated and unsaturated oligogalacturonic acids.

MCP doses used previously in pre-clinical studies *in vivo *(0.1% up to 4.0% orally administered) and human clinical studies (15 grams/day orally administered) have demonstrated an effect on both tumor growth, angiogenesis, cancer progression markers, and spontaneous metastasis [8-10,24,28,29] and the lowering of body burden of heavy metals as seen in their blood and urinary excretion levels [30,31]. These doses had an effect on galectin-3 levels in experimentally induced kidney injury which lead to reduced renal injury and fibrosis [32]. MCP concentrations used in the present investigation (up to 0.08%) are below the previously used doses in pre-clinical studies, which may be achievable and effective for modulation of immune system. However, detailed pharmacokinetic investigations of MCP by analysing the *in vivo *absorption and distribution are required to determine its effective oral dosage.

## Conclusions

The data shows MCP as a substance with immunostimulatory properties in human blood samples, including the activation of functional NK cells against K562 leukemic cells in culture. The selective immunostimulatory properties are proposed to be attributed to the presence of low degree of methyl esterification and unsaturated oligogalacturonic acids. Additional research is necessary to determine if changing the degree of esterification of oligogalactouronic acid in MCP can alter the immune response. *In vivo *studies are necessary to better understand the applications of MCP as an immune enhancer.

## List of abbreviations

DMSO: Dimethyl sulfoxide; MCP: Modified citrus pectin; NK: Natural killer; PBS: Phosphate buffered saline; SD: Standard deviation.

## Competing interests

Authors CR, AH, HC, and SJM declare that they have no competing interests.

The authors BJW and IE are employed by EcoNugenics, Inc., the makers of the MCP used in this study. IE holds patents on the method and use of MCP.

## Authors' contributions

CR designed and contributed to the interpretation of the immunological data. SJM designed, acquired data, performed analysis and interpretation of immunological data. AH designed, acquired data, performed analysis and interpretation of the molecular analysis data. HC acquired data, performed analysis and interpretation of the molecular analysis data. BJW and IE helped in the study design, data review, and interpretation of all data. All authors contributed in the drafting of the manuscript, contributing critically in its intellectual content, and have read and given their approval of the final manuscript.

## Pre-publication history

The pre-publication history for this paper can be accessed here:

http://www.biomedcentral.com/1472-6882/11/59/prepub

## References

[B1] Anderson JW Jones AE Riddell-Mason S Ten different dietary fibers have significantly different effects on serum and liver lipids of cholesterol-fed rats J Nutr 1994 124 78 83 8283297 10.1093/jn/124.1.78

[B2] Anderson JW Dietary fiber, complex carbohydrate and coronary artery disease Can J Cardiol 1995 11 55G 62G 7585294

[B3] Bingham SA Day NE Luben R Ferrari P Slimani N Norat T Clavel-Chapelon F Kesse E Nieters A Boeing H Tjønneland A Overvad K Martinez C Dorronsoro M Gonzalez CA Key TJ Trichopoulou A Naska A Vineis P Tumino R Krogh V Bueno-de-Mesquita HB Peeters PH Berglund G Hallmans G Lund E Skeie G Kaaks R Riboli E Dietary fiber in food and protection against colorectal cancer in the European Prospective Investigation into Cancer and Nutrition (EPIC): an observational study Lancet 2003 361 1496 1501 10.1016/S0140-6736(03)13174-1 12737858

[B4] Chen CH Sheu MT Chen TF Wang YC Hou WC Liu DZ Chung TC Liang YC Suppression of endotoxin induced proinflammatory responses by citrus pectin through blocking LPS signaling pathways Biochem Pharmacol 2006 72 1001 1009 10.1016/j.bcp.2006.07.001 16930561

[B5] Nangia-Makker P Conklin J Hogan V Raz A Carbohydrate-binding proteins in cancer, and their ligands as therapeutic agents Trends Mol Med 2002 8 187 192 10.1016/S1471-4914(02)02295-5 11927277

[B6] Kidd P A new approach to metastasis cancer prevention: modified citrus pectin (MCP), a unique pectin that blocks cell surface lectins Altern Med Rev 1996 1 4 10

[B7] Olano-Martin E Rimbach GH Gibson GR Rastall RA Pectin and pectic-oligosaccharides induce apoptosis in *in vivo *human colonic adenocarcinoma cells Anticancer Res 2003 23 341 346 12680234

[B8] Nangia-Makker P Hogan V Honjo Y Baccarini S Tait L Bresalier R Raz A Inhibition of human cancer cell growth and metastasis in nude mice by oral intake of modified citrus pectin J Natl Cancer Ins 2002 94 1854 1862 10.1093/jnci/94.24.1854 12488479

[B9] Guess BW Scholz MC Strum SB Lam RY Johnson HJ Jennrich RI Modified citrus pectin (MCP) increases the prostate-specific antigen doubling time in men with prostate cancer: a phase II pilot study Prostate Cancer Prostatic Dis 2003 6 301 304 10.1038/sj.pcan.4500679 14663471

[B10] Pienta KJ Naik H Akhtar A Yamazaki K Replogle TS Lehr J Donat TL Tait L Hogan V Raz A Inhibition of spontaneous metastasis in a rat prostate cancer model by oral administration of modified citrus pectin J Natl Cancer Inst 1995 87 348 353 10.1093/jnci/87.5.348 7853416

[B11] Yan J Katz A PectaSol-C modified citrus pectin induces apoptosis and inhibition of proliferation in human and mouse androgen-dependent and- independent prostate cancer cells Integr Cancer Ther 2010 9 197 203 10.1177/1534735410369672 20462856

[B12] Glinsky VV Raz A Modified citrus pectin anti-metastatic properties: one bullet multiple targets Carbohydr Res 2009 14 1788 91 10.1016/j.carres.2008.08.038 PMC2782490 19061992

[B13] Ye MB Lim BO Dietary pectin regulates the levels of inflammatory cytokines and immunoglobulins in interleukin-10 knockout mice J Agric Food Chem 2010 in press 10.1021/jf103262s 20945935

[B14] Sherry CL Kim SS Dilger RN Bauer LL Moon ML Tapping RI Fahey GC Jr Tappenden KA Freund GG Sickness behavior induced by endotoxin can be mitigated by the dietary soluble fiber, pectin, through up-regulation of IL-4 and Th2 polarization Brain Behav Immun 2010 24 631 40 10.1016/j.bbi.2010.01.015 20138982 PMC2856791

[B15] Salman H Bergman M Djaldetti M Orlin J Bessler H Citrus pectin affects cytokine production by human peripheral blood mononuclear cells Biomed Pharmacother 2008 62 579 82 10.1016/j.biopha.2008.07.058 18752921

[B16] Liu Y Ahmad H Luo Y Gardiner DT Gunasekera RS McKeehan WL Patil BS Citrus pectin: characterization and inhibitory effect on fibroblast growth factor - receptor interaction J Ag Food Chem 2001 49 3051 3057 10.1021/jf001020n 11410008

[B17] Matsumoto T Moriya M Sakurai MH Kiyohara H Tabuchi Y Yamada H Stimulatory effect of a pectic polysaccharide from a medicinal herb, the roots of *Bupleurm falcatum *L., on G-CSF secretion from intestinal epithelial cells Int Immunopharmacol 2008 8 581 588 10.1016/j.intimp.2008.01.006 18328450

[B18] Inngjerdingen M Inngjerdingen KT Patel TR Allen S Chen X Rolstad B Morris GA Harding SE Michaelsen TE Diallo D Paulsen BS Pectic polysaccharides from Biophytum petersianum Klotzsch, and their activation of macrophages and dendritic cells Glycobiology 2008 18 1074 84 10.1093/glycob/cwn090 18809620

[B19] Vos AP Haarman M van Ginkel JW Knol J Garssen J Stahl B Boehm G M'Rabet L Dietary supplementation of neutral and acidic oligosaccharides enhances Th1-dependent vaccination responses in mice Pediatr Allergy Immunol 2007 18 304 312 10.1111/j.1399-3038.2007.00515.x 17584310

[B20] Vos AP van Esch BC Stahl B M'Rabet L Folkerts G Nijkamp FP Garssen J Dietary supplementation with specific oligosaccharide mixtures decreases parameters of allergic asthma in mice Int Immunopharmacol 2007 7 1582 1587 10.1016/j.intimp.2007.07.024 17920536

[B21] Zou J Glinsky VV Landon LA Matthews L Deutscher SL Peptides specific to the galectin-3 carbohydrate recognition domain inhibit metastasis-associated cancer cell adhesion Carcinogenesis 2005 26 309 318 15528216 10.1093/carcin/bgh329

[B22] Johnson KD Glinskii OV Mossine VV Turk JR Mawhinney TP Anthony DC Henry CJ Huxley VH Glinsky GV Pienta KJ Raz A Glinsky VV Galectin-3 as a potential therapeutic target in tumors arising from malignant endothelia Neoplasia 2007 9 662 670 10.1593/neo.07433 17786185 PMC1950436

[B23] Nangia-Makker P Honjo Y Sarvis R Akahani S Hogan V Pienta KJ Raz A Galectin-3 induces endothelial cell morphogenesis and angiogensis Am J Pathol 2000 156 899 909 10.1016/S0002-9440(10)64959-0 10702407 PMC1876842

[B24] Inohara H Raz A Effects of natural complex carbohydrate (citrus pectin) on murine melanoma cell properties related to galectin-3 functions Glycoconj J 1994 11 527 532 10.1007/BF00731303 7696855

[B25] Gunning AP Bongaerts RJ Morris VJ Recognition of galactan components of pectin by galectin-3 FASEB J 2009 23 415 424 18832596 10.1096/fj.08-106617

[B26] Platt D Raz A Modulation of the lung colonization of B16-F1 melanoma cells by citrus pectin J Natl Cancer Inst 1992 84 438 442 10.1093/jnci/84.6.438 1538421

[B27] Glinsky VV Huflejt M Glinsky G Deutscher S Quinn T Effects of Thomsen-Friendenreich antigen-specific peptide p-30 on β-galactoside-mediated homotypic aggregation and adhesion to the endothelium of MDA-MB-435 human breast carcinoma cells Cancer Res 2000 60 2584 2588 10825125

[B28] Liu HY Huang ZL Yang GH Lu WQ Yu NR Inhibitory effect of modified citrus pectin on liver metastases in a mouse colon cancer model World J Gastroenterol 2008 14 7386 7391 10.3748/wjg.14.7386 19109874 PMC2778124

[B29] Azemar M Hildenbrand B Haering B Heim ME Unger C Clinical benefit in patients with advanced solid tumors treated with modified citrus pectin: a prospective pilot study Clin Med: Oncol 2007 1 73 80

[B30] Eliaz I Hotchkiss A Fishman M Rode D The effect of modified citrus pectin on urinary excretion of toxic elements Phytother Res 2006 20 859 864 10.1002/ptr.1953 16835878

[B31] Zhao ZY Liang L Fan X Yu Z Hotchkiss AT Wilk BJ Eliaz I The role of modified citrus pectin as an effective chelator of lead in children hospitalized with toxic lead levels Altern Ther Health Med 2008 14 34 38 18616067

[B32] Kolatsi-Joannou M Price KL Winyard PJ Long DA Modified citrus pectin reduces galectin-3 expression and disease severity in experimental acute kidney injury PLoS One 2011 6 e18683 10.1371/journal.pone.0018683 21494626 PMC3072992

[B33] Lim LC Fiordalisi MN Mantell JL Schmitz JL Folds JD A whole-blood assay for qualitative and semiquantitative measurements of CD69 surface expression on CD4 and CD8 T lymphocytes using flow cytometry Clin Diagn Lab Immunol 1998 5 392 398 9605996 10.1128/cdli.5.3.392-398.1998 PMC104529

[B34] Liu L Chahroudi A Silvestri G Wernett ME Kaiser WJ Safrit JT Komoriya A Altman JD Packard BZ Feinberg MB Visualization and quantification of T cell-mediated cytotoxicity using cell-permeable fluorogenic caspase molecules Nat Med 2000 8 185 189 10.1038/nm0202-185 11821904

[B35] Jerome KR Sloan DD Aubert M Measuring T-cell mediated cytotoxicity using antibody to activated caspases Nat Med 2002 9 4 5 10.1038/nsb0102-4 12514701

[B36] Hotchkiss AT Hicks KB Analysis of pectate lyase-generated oligogalacturonic acids by high-performance anion-exchange chromatography and pulsed amperometric detection Carbohydr Res 2003 247 1 7 10.1016/s0008-6215(01)00170-7 11502269

[B37] Robertson MJ Ritz J Biology and clinical relevance of natural killer cells Blood 1990 76 2421 2438 2265240

[B38] Takeda K Okumura K CAM and NK Cells eCAM 2010 1 17 27 10.1093/ecam/neh014 PMC442116 15257322

[B39] Fanaro S Jelinek J Stahl B Boehm G Kock R Vigi V Acidic oligosaccharides from pectin hydrolysate as new component for infant formulae: Effect on intestinal flora, stool characteristics, and pH J Pediatr Gasteroentero Nutrit 2005 41 186 190 10.1097/01.mpg.0000172747.64103.d7 16056097

